# Ginsenoside Rb2 Alleviated Atherosclerosis by Inhibiting M1 Macrophages Polarization Induced by MicroRNA-216a

**DOI:** 10.3389/fphar.2021.764130

**Published:** 2022-01-03

**Authors:** Shuting Wang, Shujun Yang, Yu Chen, Yutong Chen, Rongxia Li, Shuang Han, Adalaiti Kamili, Yiyi Wu, Weili Zhang

**Affiliations:** ^1^ State Key Laboratory of Cardiovascular Disease, FuWai Hospital, National Center for Cardiovascular Diseases, Peking Union Medical College and Chinese Academy of Medical Sciences, Beijing, China; ^2^ Department of Geriatric Medicine, Xiangya Hospital, Central South University, Changsha, China; ^3^ Xiamen Cardiovascular Hospital, Xiamen University, Xiamen, China

**Keywords:** atherosclerosis, macrophage, ginsenoside Rb2, microRNA-216a, lipid

## Abstract

**Introduction:** Atherosclerosis is a chronic disease characterized by the inflammatory process and lipid depositions. We previously reported that microRNA-216a (miR-216a) can accelerate the progression of atherosclerosis by promoting the polarization of M1 pro-inflammatory phenotype. Ginsenoside Rb2 (Rb2), the major pharmacologically active compound extracted from ginseng, has a high affinity to miR-216a. In this study, we aimed to investigate whether Rb2 can counteract the effect of miR-216a in macrophages to ameliorate atherosclerosis.

**Methods:** The apolipoprotein E deficiency (ApoE^−/−^) mice model was chronically infected with miR-216a adenovirus via the tail vein and then intraperitoneally injected with Rb2. The plaque lesion area and stability of thoracic aorta were examined. The human myeloid leukemia mononuclear cells (THP-1) or human peripheral blood mononuclear cells (PBMCs) were cultured *in vitro*, transfected with miR-216a mimics, and treated with Rb2 to explore the mechanisms of Rb2 on the polarization of M1 macrophages, inflammatory process, and lipid accumulation.

**Results:** In the atherosclerotic ApoE^−/−^ mice model, miR-216a greatly increased en face aortic lesion area of the thoracic aorta, lipid accumulation, and M1 macrophages infiltration in plaques, whereas these effects of miR-216a on atherosclerosis burden were significantly alleviated by Rb2 treatment. In the *in vitro* THP-1 model, the flow cytometry experiment showed that Rb2 treatment inhibited miR-216a–mediated polarization of M1 macrophages characterized by the surface marker CD86 expression but had no effects on M2 polarization characterized by the surface marker CD206 expression. Mechanistically, Rb2 suppressed the miR-216a–mediated inflammatory response through the Smad3/nuclear factor kappa B inhibitor alpha pathway. Moreover, Rb2 reduced the lipid uptake and promoted cholesterol efflux by counteracting the effects of miR-216a in the THP-1–derived foam cells and in the PBMC-derived foam cells under the oxidized low-density lipoproteins.

**Conclusion:** Our findings indicated that Rb2 might be a potential therapeutic molecule for atherosclerosis by attenuating the atherosclerosis plaque lesion, lipid accumulation, and M1 macrophages polarization by targeting miR-216a. Given that accumulation of foam cells in the intima takes place chronically, the role of Rb2 in atherosclerosis progression needs further investigation.

## Introduction

Cardiovascular diseases contribute to the global burden of morbidity and mortality. Atherosclerosis is a chronic inflammatory disorder disease which underlies the pathogenesis of cardiovascular diseases. As we know, the monocytes/macrophages are well recognized as the central participant in the inflammatory process during atherosclerosis development (Tabas and Bornfeldt, 2016). The polarized M1 pro-inflammatory phenotype can produce inflammatory cytokines, promote excessive lipid accumulation and formation of foam cells under the oxidatively modified low-density lipoproteins (ox-LDLs). The inhibition of macrophages polarization may be a potential therapeutic strategy for treating atherosclerosis.

MicroRNAs (miRNAs) are small single-stranded noncoding molecules with approximately 22 nucleotides in length, playing a critical role in regulating gene expression via combination with the 3′-untranslated region (3′-UTR) of the mRNA sequence. Several studies have shown that miRNAs can regulate the process of macrophage inflammation and lipid phagocytosis. Qi et al. (2020) found that miR-520a-3p inhibits macrophage polarization and promotes the development of atherosclerosis via targeting the UV radiation resistance–associated gene in apolipoprotein E deficiency (ApoE^−/−^) mice. Xu et al. (2020) reported that the ablation of miR-34a increases cholesterol efflux and cholesterol transport in macrophages by modulating ATP-binding cassette subfamily A member 1 (ABCA1) and ATP-binding cassette subfamily G member 1 (ABCG1). Our previous studies found that a senescence-related miRNA, miR-216a, increases in patients with atherosclerotic vulnerable plaque and promotes instability of atherosclerotic plaque in the ApoE^−/−^ mice model. Moreover, miR-216a can promote M1 macrophage polarization, lipid accumulation, and cellular senescence by activating telomerase through the Smad3/nuclear factor kappa B (NF-κB) signaling pathway (Yang et al., 2019). These data suggested that miR-216a may be a potential therapeutic target for treating atherosclerosis.

Ginsenoside Rb2 (Rb2), a 20 (S)-protopanaxadiol glycoside, is the main bioactive component of ginseng. Rb2 has various kinds of biological effects. For example, Rb2 can inhibit lipopolysaccharide (LPS)–induced inflammatory response by promoting G protein–coupled receptor 120 (GPR120) expression in mouse macrophage RAW264.7 cells (Huang et al., 2017). Hong et al. (2019) reported that Rb2 reduces the weight of obese mice and decreases lipid accumulation in adipose tissues. However, the effects of Rb2 on atherosclerosis and plaque stability have not been reported.

Our recent work has shown that Rb2 has a high affinity to miR-216a via bioinformatics analysis and microscale thermophoresis experiment (Chen et al., 2021). In the present study, we aimed to examine whether Rb2 can counteract the role of miR-216a in the inflammatory response, macrophages polarization, and lipid phagocytosis in atherosclerotic ApoE^−/−^ mice *in vivo* and in macrophages or foam cells *in vitro*, expecting to find a novel therapeutic strategy for atherosclerosis.

## Methods and Materials

### Animal Models With miR-216a Infection and Rb2 Treatment

An ApoE^−/−^ atherosclerotic mouse model was established. The male ApoE^−/−^ mice on a C57BL/6J background (Vital River Laboratory Animal Technology, Beijing, China) were fed Western diet for 11 weeks. At 8 weeks, the mice were randomly divided into four groups and received the corresponding treatments for 3 weeks: adenovirus negative control group (Ad-NC) (*n* = 8), adenovirus miR-216a group (Ad-miR-216a) (*n* = 7), Ad-NC + Rb2 group (*n* = 8), and Ad-miR-216a + Rb2 group (*n* = 8). The mice in the Ad-miR-216a and Ad-miR-216a + Rb2 groups were injected with miR-216a recombinant adenovirus (Weizhen Biosciences, Jinan, China) at the dose of 2 × 10^9^ units via the tail vein. After 3 days, the mice in the Ad-NC + Rb2 and Ad-miR-216a + Rb2 groups were intraperitoneally injected with 20 mg/kg of Rb2 (MedChemExpress, Monmouth, NJ, United States) once every 2 days. The mice in the Ad-NC and Ad-miR-216a groups received saline of the same volume.

At the end of the study, the mice were euthanized by a mixture of ketamine (100 mg/kg) and xylazine (10 mg/kg), and then, the aortas and peripheral blood were collected. The plasma was separated by centrifugation 2000 rpm and stored at −80°C until use. The plasma levels of cholesterol and triglyceride were measured by the Labospect 008 AS Automatic Biochemical Analyzer (Hitachi, Tokyo, Japan). All animal handling and procedures were approved by the Institutional Animal Care and Use Committee at the FuWai Hospital.

### En Face Analysis of the Aorta in Mice

The aortas of ApoE^−/−^ mice were collected to analyze the status of atherosclerosis plaque. The Oil red O (Sigma-Aldrich, Saint Louis, MO, United States) staining method was used to assess en face lesions of the aortas. The images were captured with a stereomicroscope-dedicated camera (Zeiss, Jena, Germany) and analyzed with Image-pro Plus 6.0. The extent of the lesion area is the percentage of the total area of the aorta that was covered by lesions.

### Histology and Immunohistochemical Analyses

Hematoxylin–eosin (HE) staining was performed on 8-μm-thick frozen sections for histological analysis to observe the plaque lesion area. Masson’s trichrome staining (Leagene Biotechnology, Beijing, China) was used to examine collagen content of plaque. Oil red O staining was used to examine the accumulation of neutral lipids in the plaque area. Immunohistochemical staining was used to assess plaque macrophages and matrix metalloproteinase 9 (MMP-9) expression. The details are described in the Supplementary Materials.

### Cell Culture, miRNA Transfection, and Rb2 Treatment

The human acute monocytic leukemia cell line, THP-1 cells (China Infrastructure of Cell Line Resources, Beijing, China), were cultured in the RPMI 1640 medium (HyClone, Logan, UT, United States) with 10% fetal bovine serum (Sigma-Aldrich, Saint Louis, MO, United States) in a 5% CO_2_ incubator at 37°C. The THP-1 cells were differentiated to macrophages under the stimulation of 100 ng/ml phorbol-12-myristate-13-acetate (PMA) (Sigma-Aldrich, Saint Louis, MO, United States) for 48 h. The miR-216a mimics or negative control were transfected into macrophages at a final concentration of 50 nM using lipofectamine 3000 reagent (Invitrogen, Carlsbad, CA, United States) for 8 h. Then, the cells were treated with 10 μM Rb2 (MedChemExpress, Monmouth, NJ, United States) for 48 h. The sequence of miR-216a mimics was designed as the following: sense 5′-UAA​UCU​CAG​CUG​GCA​ACU​GUG​A-3′ and antisense 5′-ACA​GUU​GCC​AGC​UGA​GAU​UAU​U-3′.

The human peripheral blood was obtained from healthy volunteers with informed consent. The peripheral blood mononuclear cells (PBMCs) were isolated using a Histopaque®-1077 density gradient (Sigma-Aldrich, Saint Louis, MO, United States) as described previously (Hirose et al., 2011). The monocytes were differentiated into macrophages by incubation with RPMI 1640 medium containing 20% fetal bovine serum and 100 ng/mL M-CSF (PeproTech, Suzhou, China) for 7 days. Subsequently, macrophages were transfected with miR-216a mimics for 8 h and treated with 10 μM Rb2 for 36 h, then transformed to foam cells under the stimulation of 50 μg/ml ox-LDLs for 12 h.

### Dual-Luciferase Reporter Assay

The Dual-Luciferase Reporter Assay was used to assess the effects of Rb2 on the expression of Smad3, the direct target of miR-216a. The pMIR-REPORT-Smad3-3′UTR luciferase plasmid was constructed according to our previous study (Yang et al., 2018). The details are shown in the Supplementary Materials.

### Telomerase Enzyme Activity Measurement

The telomerase repeat amplification protocol assay was performed to assess the telomerase activity during macrophages differentiation and polarization (Hou et al., 2001; Yang et al., 2019), with details as shown in the Supplementary Materials.

### Real-Time Quantitative PCR Assay and Western Blot Analysis

The mRNAs and miRNAs expression were analyzed by real-time quantitative (RT-q) PCR on the ABI 7500 System (Applied Biosystems, Foster City, CA, United States), and the gene primers are shown in Supplementary Table S1. The effects of Rb2 on protein expression of Smad3 and nuclear factor kappa B inhibitor alpha (IκBα) in macrophages were determined by the western blot assay. The details are shown in the Supplementary Materials.

### Assays for Lipid Uptake Abilities and Cholesterol Efflux of Foam Cells

The effects of Rb2 on lipid uptake ability of THP-1–derived foam cells or PBMC-derived foam cells were examined using Oil red O staining. The cholesterol efflux assay was performed in macrophages incubated with [^3^H]-cholesterol to explore the effect of Rb2 in cholesterol transport. The details are shown in the Supplementary Materials.

### Flow Cytometry Assay

The effects of Rb2 on miR-216a–mediated polarization of M1 macrophages were assessed by flow cytometry, and the surface markers CD86 of M1 macrophages and CD206 of M2 macrophages were examined. Cell apoptosis and death were detected by flow cytometry with the Annexin V/PI assay with the FITC Annexin V/PI Apoptosis kit (BD Biosciences, New Jersey, United States). The details are shown in the Supplementary Materials.

### Senescence-Associated β-Galactosidase Staining

Macrophages were transfected with miR-216a mimics and treated with Rb2 as described above. The senescent status of the macrophages was assessed by *in situ* staining for senescence-associated β-galactosidase (SA-β-gal). The details are described in the Supplementary Materials.

### Statistical Analysis

Data analysis was performed using SPSS 20.0 statistical software (SPSS Inc., Chicago, United States). Quantitative variables are presented as mean ± SEM. The statistical significance of differences was calculated using one-way ANOVA, and a *p* value < 0.05 was considered statistically significant.

## Results

### Rb2 Inhibited the Atherosclerotic Plaque Development Induced by miR-216a in Mice

**FIGURE 1:**
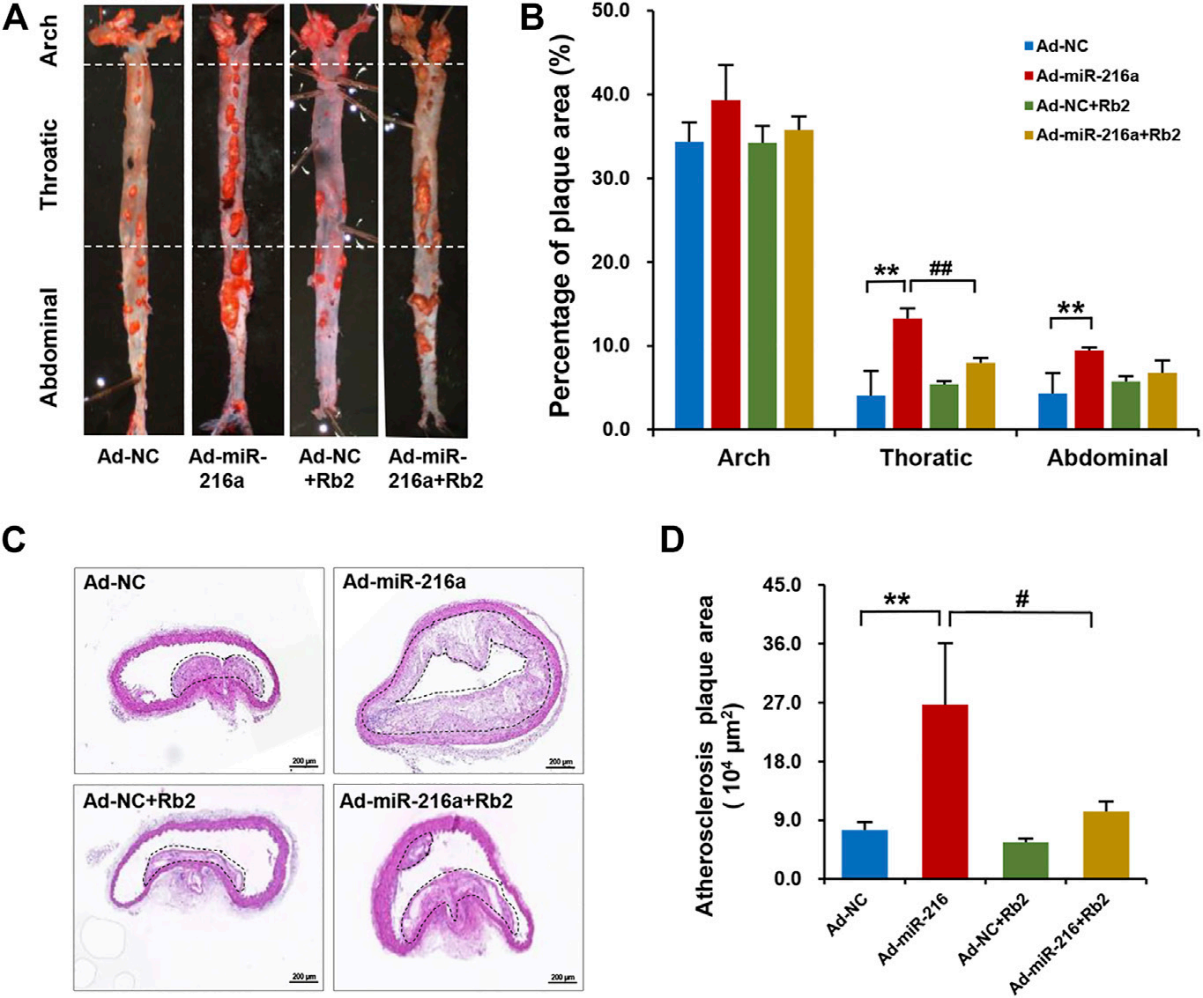
Rb2 inhibited the atherosclerosis plaque development induced by miR-216a in mice. **(A)** Representative image of lesions in en face aorta evaluated by Oil red O staining. **(B)** Quantification of Oil red O–positive areas in the aortas cut lengthwise. **(C)** Representative image of HE staining in the thoracic aorta from the Ad-NC group, Ad-miR-216a group, Ad-NC + Rb2 group, and Ad-miR-216a + Rb2 group. Scale bar = 50 μM. **(D)** Quantification of the lesion area of the thoracic aorta from the Ad-NC group, Ad-miR-216a group, Ad-NC + Rb2 group, and Ad-miR-216a + Rb2 group. **p < 0.01, compared to the Ad-NC group; #*p* < 0.05, ##*p* < 0.01, compared to the Ad-miR-216a group. *n* = 5 for each group.

To explore the effect of Rb2 on atherosclerosis plaque induced by miR-216a *in vivo*, an ApoE^−/−^ atherosclerotic mice model was established. The analyses of atherosclerosis lesion formation on the aortas cut lengthwise revealed that miR-216a increased en face the aortic lesion area of the thoracic aorta by 200% compared with the control group, whereas the effect of miR-216a on atherosclerosis burden was significantly decreased by 45% with Rb2 treatment (Figures 1A,B). Therefore, the thoracic aorta was chosen for further experiments to assess the stability of plaque. HE staining of the frozen sections in the thoracic aorta showed that miR-216a increased the lesion area by 300% compared with the control group, and this effect also diminished by 74% with Rb2 treatment (Figures 1C,D).

### Rb2 Reduced Macrophages Infiltration and Polarization in Plaque Lesions

**FIGURE 2:**
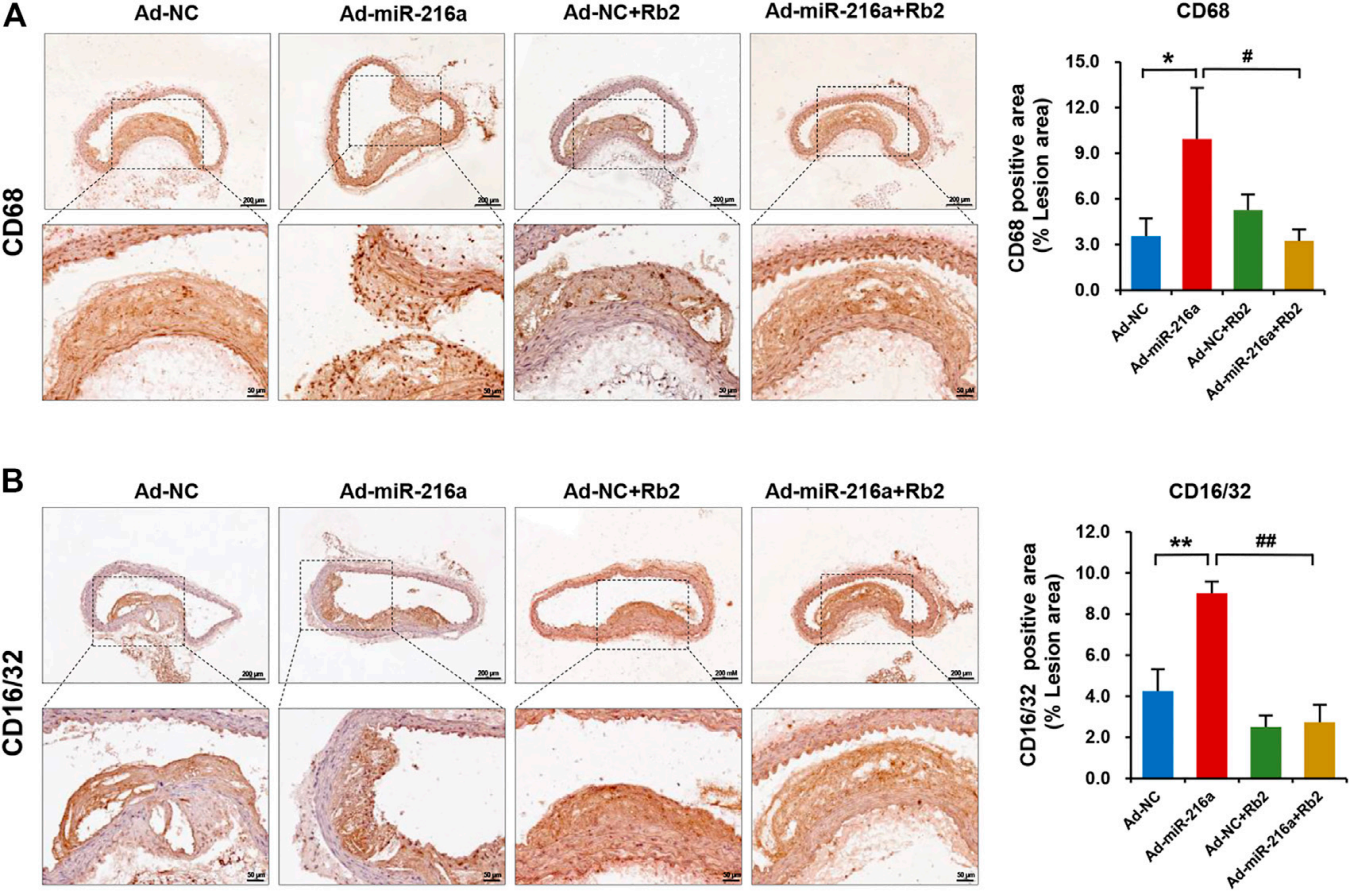
Rb2 reduced macrophages infiltration and polarization in plaque in mice. **(A)** Representative image and quantification of immunohistochemistry staining of CD68 in the thoracic aorta from the Ad-NC group, Ad-miR-216a group, Ad-NC + Rb2 group, and Ad-miR-216a + Rb2 group. **(B)** Representative image and quantification of immunohistochemistry staining of CD16/32 in the thoracic aorta from the Ad-NC group, Ad-miR-216a group, Ad-NC + Rb2 group, and Ad-miR-216a + Rb2 group. *p < 0.05, **p < 0.01, compared to the Ad-NC group. #*p* < 0.05, ##*p* < 0.01, compared to the Ad-miR-216a group. *n* = 5 for each group. Scale bar = 200 μM or 50 μM.

Next, we investigated whether macrophages polarization was involved in the role of Rb2 in atherosclerosis progression. The immunohistochemistry staining showed that intima macrophages stained with surface marker CD68 were markedly increased by 200% in atherosclerotic plaque of the Ad-miR-216a group compared with the control group, while the number of infiltrated macrophages was reduced in the Ad-miR-216a + Rb2 group (Figure 2A). Moreover, staining of M1 marker CD16/CD32 showed that pro-inflammatory M1 subsets were dramatically increased by 150% in plaque areas in the Ad-miR-216a group, which was also suppressed by Rb2 treatment (Figure 2B).

### Rb2 Inhibited Lipid Accumulation in Plaque Lesions

**FIGURE 3:**
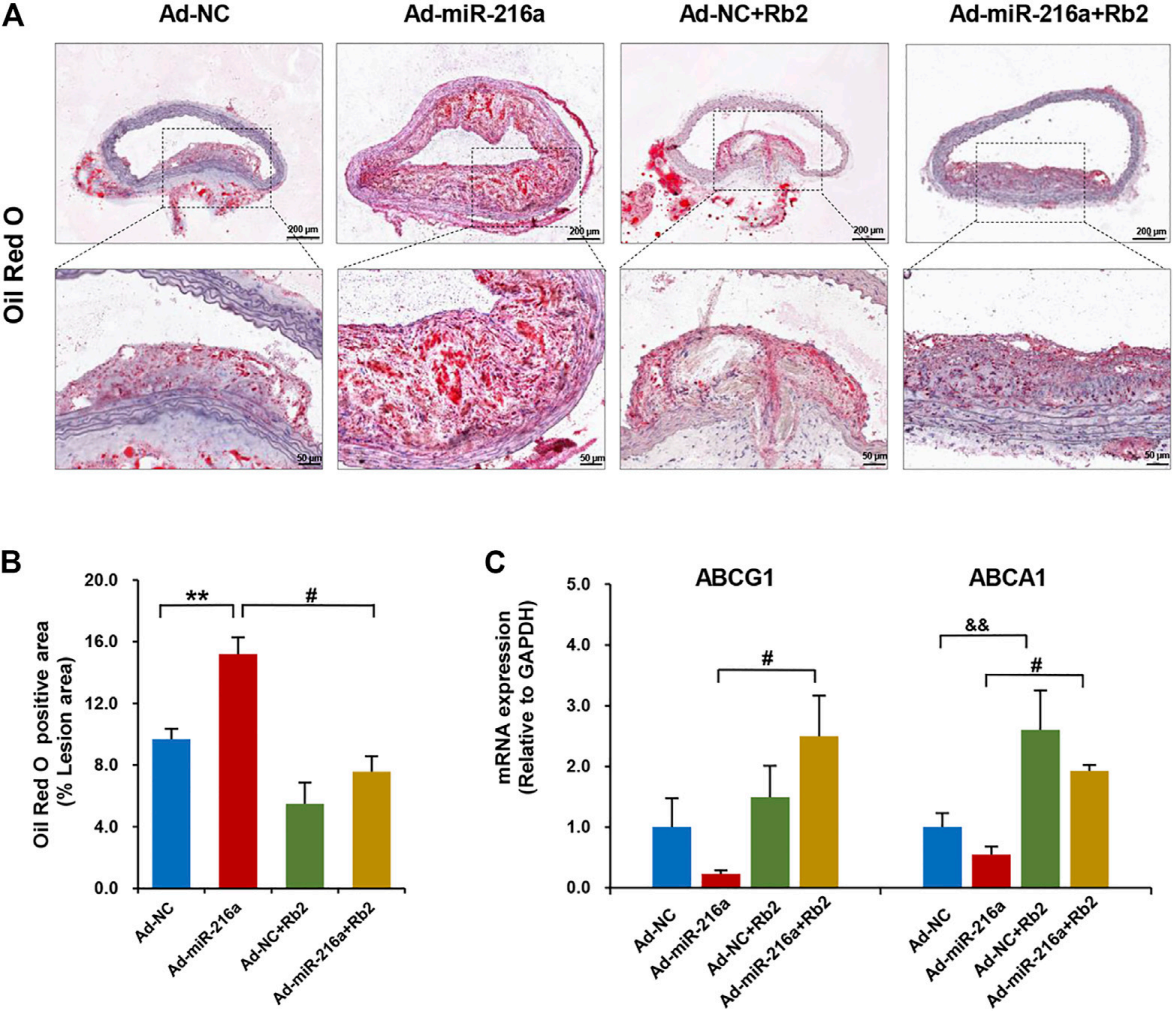
Rb2 inhibited lipid accumulation in plaque and peritoneal macrophages of mice. **(A)** Representative image of Oil red O staining in the thoracic aorta from the Ad-NC group, Ad-miR-216a group, Ad-NC + Rb2 group, and Ad-miR-216a + Rb2 group. Scale bar = 200 μM or 50 μM. **(B)** Quantification of the percentage of lipid (of the total lesion area) in the thoracic aorta from the Ad-NC group, Ad-miR-216a group, Ad-NC + Rb2 group, and Ad-miR-216a + Rb2 group. **(C)** The mRNA expression of ABCG1 and ABCA1 of peritoneal macrophages from the Ad-NC group, Ad-miR-216a group, Ad-NC + Rb2 group, and Ad-miR-216a + Rb2 group. **p < 0.01, &&*p* < 0.01, compared to the Ad-NC group. #*p* < 0.05, compared to the Ad-miR-216a group. *n* = 5 for each group.

The miR-216a led to an increase of lipid accumulation by 50% in plaques of the thoracic aorta when compared with the control group, whereas Rb2 treatment alleviated lipid accumulation by 55% in the Ad-miR-216a + Rb2 group (Figures 3A,B). In addition, the decreased mRNA expression of cholesterol efflux–related genes ABCG1 and ABCA1 induced by miR-216a were restored by Rb2 treatment in peritoneal macrophages in the ApoE^−/−^ atherosclerotic mice model (Figure 3C). However, either miR-216a or Rb2 has no significant effect on the serum lipid profiles of mice (Supplementary Table S2).

### Rb2 Improved Plaque Stability by Increasing the Collagen Content in Plaque Lesions

**FIGURE 4:**
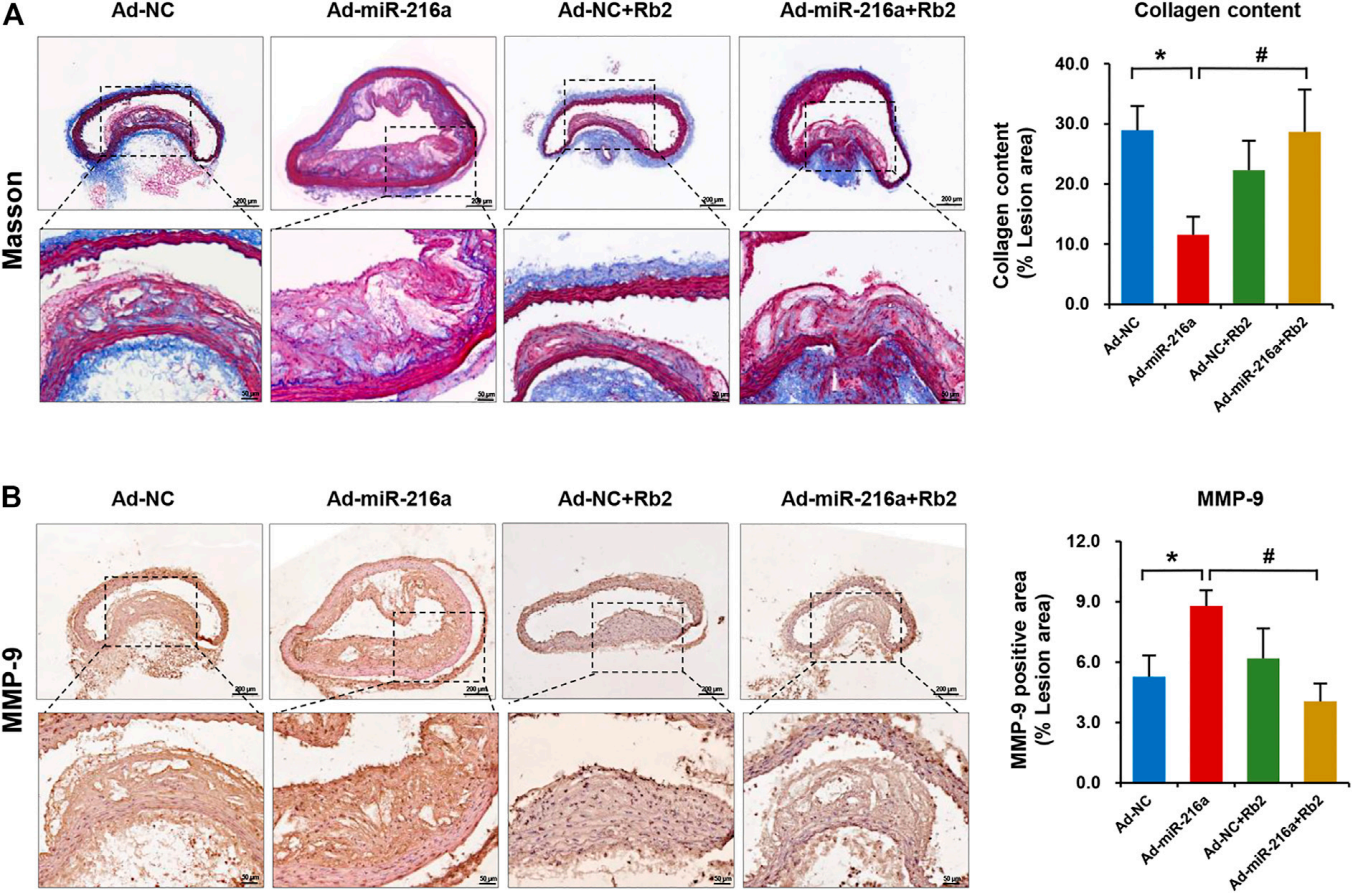
Rb2 increased the collagen content in plaque in mice. **(A)** Representative image of Masson's trichrome staining and quantification of the percentage of collagen (of the total lesion area) in the thoracic aorta from the Ad-NC group, Ad-miR-216a group, Ad-NC + Rb2 group, and Ad-miR-216a + Rb2 group. **(B)** Representative image and of quantification immunohistochemistry staining of MMP-9 in the thoracic aorta from the Ad-NC group, Ad-miR-216a group, Ad-NC + Rb2 group, and Ad-miR-216a + Rb2 group. *p < 0.05, compared to the Ad-NC group. ^#^ *p* < 0.05, compared to the Ad-miR-216a group. *n* = 5 for each group. Scale bar = 200 μM or 50 μM.

MMPs are mainly secreted by M1 macrophages and MMP-9 is a crucial factor to degrade collagen in plaques (Newby, 2014). To further explore the impacts of Rb2 on atherosclerosis plaque stability, the collagen deposition of plaque was assessed by Masson’s trichrome staining. The collagen content of thoracic aorta lesion was decreased by 62% in the Ad-miR-216a group compared with the control group, while it was increased by 250% after Rb2 treatment (Figure 4A). The percentage of MMP-9–positive area in the thoracic aorta plaques was significantly reduced by 50% in the Ad-miR-216a + Rb2 group when compared with the Ad-miR-216a group (Figure 4B).

### Rb2 Suppressed Macrophage Polarization by miR-216a/Smad3/IκBα Pathway *in Vitro*

**FIGURE 5:**
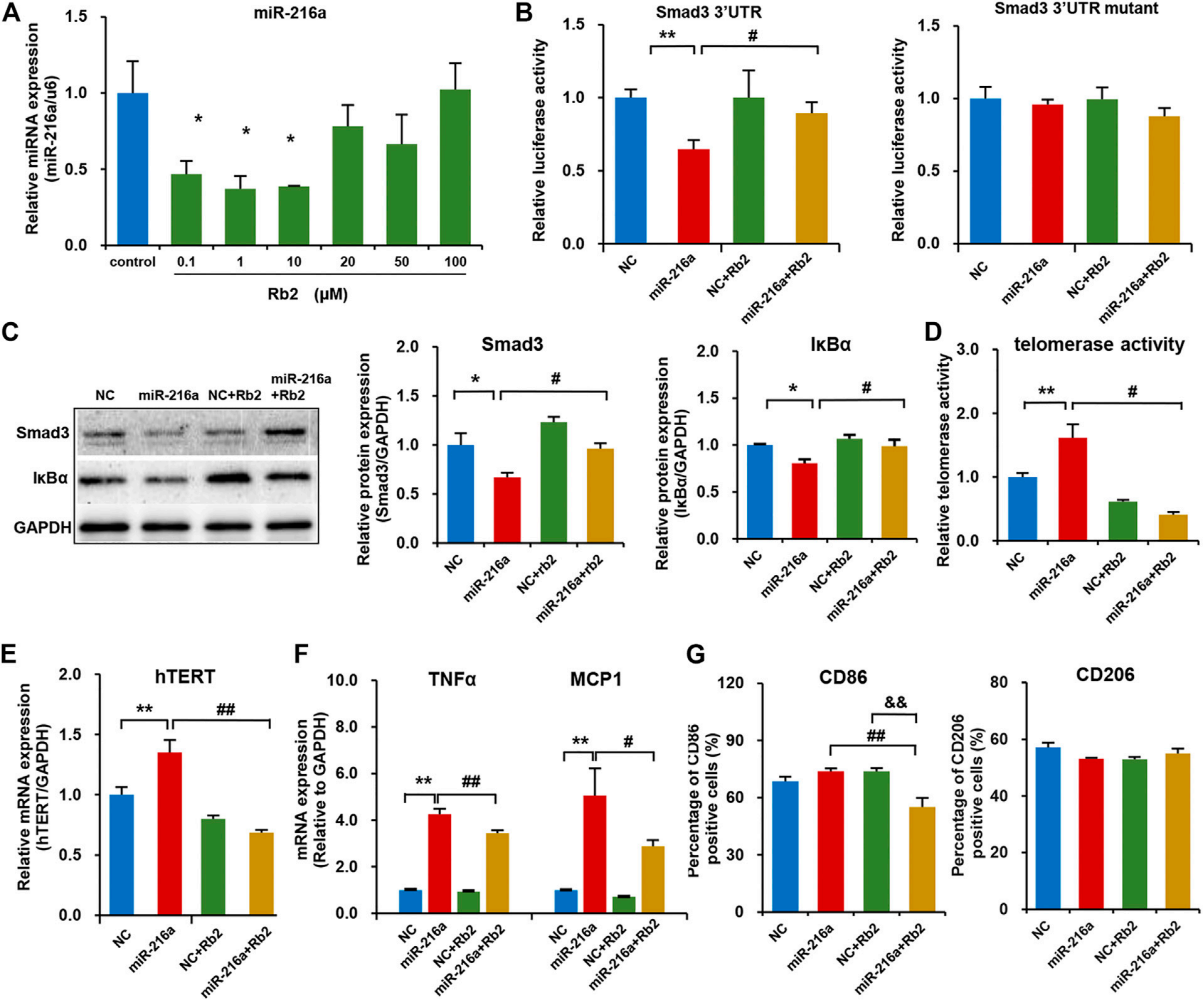
Rb2 suppressed macrophage polarization in the THP-1 model by the miR-216a/Smad3/IκBα pathway. **(A)** The expression of miR-216a in M1 macrophages treated with serial concentrations of 0.1, 1, 10, 20, 50, and 100 μM of Rb2. **(B)** The effects of Rb2 on luciferase activity of HEK293T cells when co-transfected with miR-216a and Smad3-3′UTR or mutant luciferase reporter vector (*n* = 8 for each group). **(C)** The protein expression of Smad3 and IκBα with or without Rb2 treatment in macrophages transfected with miR-216a mimics. **(D)** The telomerase activity of macrophages with or without Rb2 treatment in macrophages transfected with miR-216a mimics. **(E)** The mRNA expression of hTERT with or without Rb2 treatment in macrophages transfected with miR-216a mimics. **(F)** The mRNA expression of TNFα and MCP-1 with or without Rb2 treatment in macrophages transfected with miR-216a mimics. **(G)** The expression of surface markers CD86 (labelled M1 macrophages) and CD206 (labelled M2 macrophages) with or without Rb2 treatment in macrophages transfected with miR-216a mimics. *p < 0.05, **p < 0.01, ^&&^ *p* < 0.01 compared to the control, NC, or NC + Rb2 group. ^#^ *p* < 0.05, ^##^ *p* < 0.01, compared to the miR-216a group. *n* = 5 for each group.

Our previous study reported that miR-216a can activate telomerase activity and promote the polarization of pro-inflammatory M1 phenotype via Smad3/IκBα signaling (Yang et al., 2019). In this study, Rb2 treatment with concentrations of 0.1, 1, or 10 μM significantly decreased endogenous miR-216a expression in macrophages by 53, 63 and 62%, respectively (Figure 5A). Given that the Kd value of Rb2 binding to miR-216a was 17.6 μM in the microscale thermophoresis experiment from our previous study (Chen et al., 2021), the concentration of 10 μM of Rb2 was chosen as the optimal dose for experiments.

In the luciferase assay in HEK293T cells, miR-216a reduced the luciferase activity of pMIR-Smad3-3′UTR reporter by targeting the Smad3, and this effect was counteracted by Rb2 treatment (Figure 5B). As for the pMIR-Smad3-3′UTR mutant reporter, unsurprisingly, there were no changes in luciferase activity when treating with miR-216a or Rb2 as compared to the control group. Moreover, western blot showed that Rb2 treatment restored the Smad3 and IκBα protein levels in macrophages with miR-216a overexpression (Figure 5C).

Next, the telomerase activity in macrophages was assessed with or without Rb2 treatment. miR-216a increased the telomerase activity by 61%, and Rb2 suppressed the effect of miR-216a on telomerase activity by 75% (Figure 5D). Consistently, Rb2 downregulated the mRNA expression of human telomerase reverse transcriptase (hTERT) induced by miR-216a (Figure 5E).

The effect of Rb2 on macrophages polarization was assessed. miR-216a promoted M1 macrophages polarization characterized by the expression of inflammatory markers including monocyte chemotactic protein-1 (MCP-1) and tumor necrosis factor alpha (TNFα), while this effect was ameliorated by Rb2 treatment (Figure 5F). Furthermore, flow cytometry experiment showed that Rb2 treatment inhibited the miR-216a–mediated polarization of M1 macrophages characterized by the surface marker CD86 expression but had no effects on M2 polarization characterized by the surface marker CD206 expression (Figure 5G).

Finally, the flow cytometry assay showed that the percentages of dead cells in THP-1 PMA stimulation and miR-216a transfection were all less than 5% (Supplementary Figure S1), indicating a low toxic effect of PMA and lipofectamine reagents on THP-1 cells. Moreover, the cytotoxic effect of Rb2 on foam cells was assessed, and there was no significant impact on cell death in THP-1–derived foam cells (Supplementary Figure S2).

### Rb2 Alleviated miR-216a–Induced Lipid Accumulation in Foam Cells

**FIGURE 6:**
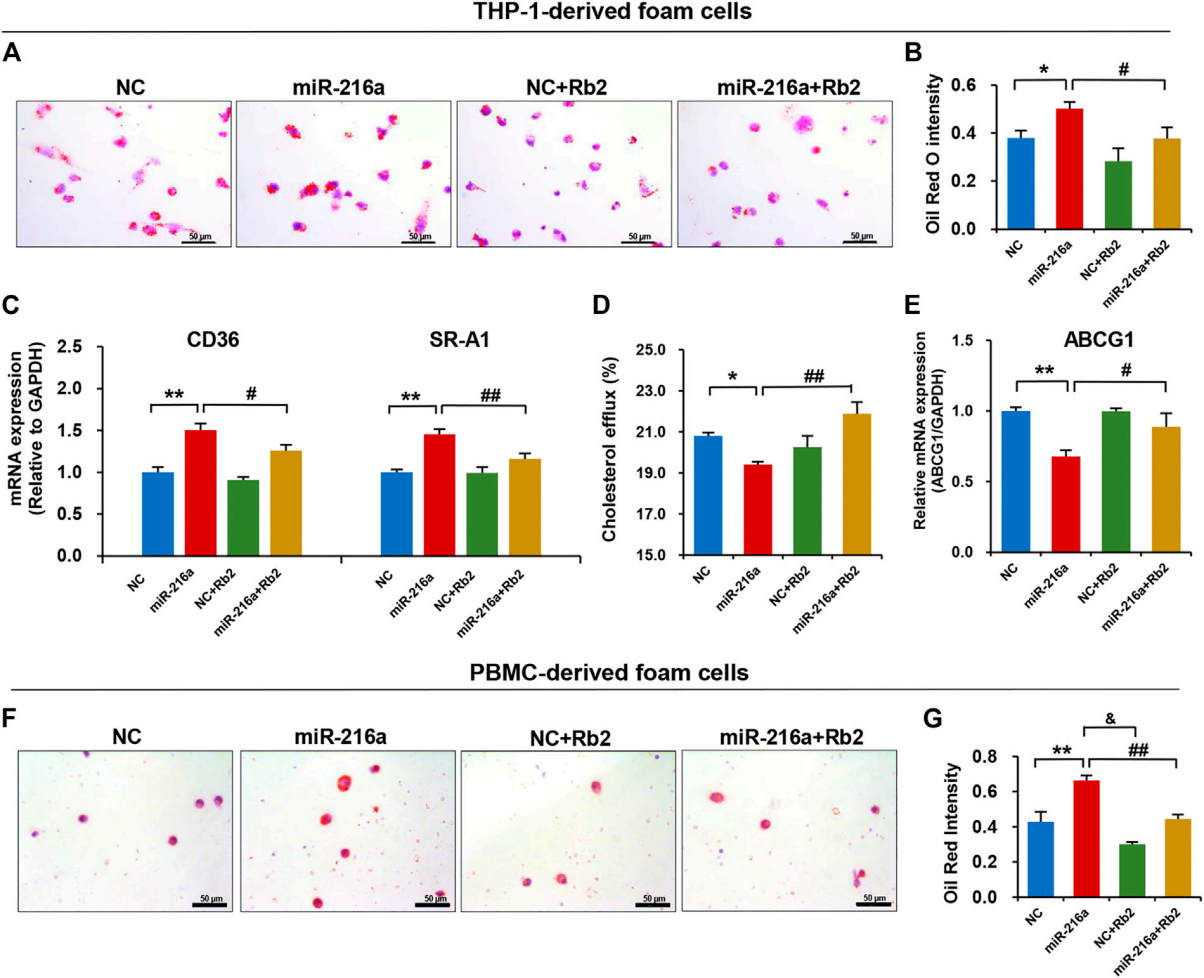
Rb2 attenuated miR-216a–induced lipid accumulation in foam cells. **(A)** Photomicrographs of lipid accumulation within the cytoplasm (red staining) with or without Rb2 treatment in THP-1–derived foam cells transfected with miR-216a mimics. **(B)** Quantification of the percentage of Oil red O–positive area (of the total cell area) with or without Rb2 treatment in THP-1–derived foam cells transfected with miR-216a mimics. **(C)** The mRNA expression of lipid uptake–related genes CD36 and SR-A1 with or without Rb2 treatment in THP-1–derived foam cells transfected with miR-216a mimics. **(D)** The cholesterol efflux ability with or without Rb2 treatment in THP-1-derived macrophages transfected with miR-216a mimics. **(E)** The mRNA expression of cholesterol efflux gene ABCG1 with or without Rb2 treatment in THP-1–derived foam cells transfected with miR-216a mimics. **(F)** Photomicrographs of lipid accumulation within the cytoplasm (red staining) with or without Rb2 treatment in the human PBMC–derived foam cells transfected with miR-216a mimics. **(G)** Quantification of the percentage of Oil red O–positive area (of the total cell area) with or without Rb2 treatment in the human PBMC-derived foam cells transfected with miR-216a mimics. *p < 0.05, **p < 0.01, compared to the NC group. ^&^ *p* < 0.05, ^#^ *p* < 0.05, ^##^ *p* < 0.01, compared to the miR-216a group. *n* = 5 for each group. Scale bar = 50 μM.

We tested the effect of Rb2 on lipid accumulation in foam cells derived from THP-1 cells and human PBMC. The Oil red O staining showed that the lipid uptake ability of THP-1–derived foam cells was significantly increased in the miR-216a overexpression group but was decreased by 34% when treated with Rb2 (Figures 6A,B). In addition, the elevated expression of CD36 and macrophage scavenger receptor 1 (SR-A1) induced by miR-216a were also reduced after Rb2 treatment (Figure 6C).

By contrast, the cholesterol efflux of macrophages was reduced by 13% in the miR-216a overexpression group but was increased when treated with Rb2 (Figure 6D). The expression of cholesterol efflux–related gene ABCG1 induced by miR-216a was reversed by 30% after Rb2 treatment (Figure 6E).

Given that primary monocytes or macrophages are an adequate model to study plaque macrophages, the human PBMCs were cultured and then transformed to foam cells by ox-LDL. The lipid accumulation in foam cells was increased by miR-216a, while this effect was suppressed by Rb2 treatment, indicating that Rb2 alleviated miR-216a–mediated lipid deposition in foam cells (Figures 6F,G).

### Rb2 Attenuated miR-216a–Induced Cell Senescence in Macrophages

**FIGURE 7:**
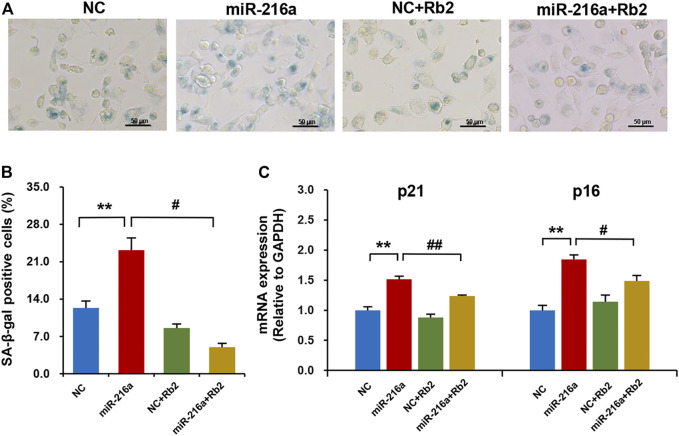
Rb2 attenuated miR-216a–induced cell senescence in macrophages. **(A)** Photomicrographs of senescent cells characterized by accumulation of senescence-associated β-galactosidase (SA-β-gal) within the cytoplasm (blue staining) with or without Rb2 treatment in macrophages transfected with miR-216a mimics. Scale bar = 50 μM. **(B)** Quantification of the percentage of SA-β-gal–positive cells (of the total cell number) with or without Rb2 treatment in macrophages transfected with miR-216a mimics. **(C)** The mRNA expression of senescent-related genes p53 and p21 with or without Rb2 treatment in macrophages transfected with miR-216a mimics. **p < 0.01, compared to the NC group. ^#^ *p* < 0.05, ^##^ *p* < 0.01, compared to the miR-216a group. *n* = 5 for each group.

The SA-β-gal staining showed that miR-216a promoted the macrophage senescence, and this effect was reduced by 75% after Rb2 treatment (Figures 7A,B). Consistently, the elevated expression of aging-related genes p21 and p16 induced by miR-216a was restored with Rb2 treatment (Figure 7C).

In addition, cell apoptosis and necrosis were examined. MiR-216a inhibited apoptosis in macrophages by 31%, while Rb2 had no effect on apoptosis (Supplementary Figure S3A). Cell necrosis of macrophages was not affected by miR-216a or Rb2 (Supplementary Figure S3B).

## Discussion

In this study, our data firstly demonstrated that ginsenoside Rb2 reduced macrophages infiltration and pro-inflammatory M1 phenotype polarization, inhibited lipid accumulation, increased the collagen content in plaque lesions, and thus alleviated the plaque instability and atherosclerosis progression through neutralizing the effects of miR-216a in the ApoE^−/−^ atherosclerotic mice model. On the mechanism, Rb2 exerted the inhibitory role in M1 macrophages polarization, inflammatory response, and lipid accumulation via miR-216a–mediated Smad3/IκBα signaling pathway.

miRNAs are emerging as potential targets for drug discovery, such as oligonucleotides that are complementary to miRNA and block its activity, duplex or chemically modified single-stranded RNAs that mimic miRNA and trigger enhanced activity (Dangwal and Thum, 2014). However, there are some drawbacks, for example, unwanted exogenous RNA immunogenicity, poor stability, weak cell permeability, and high costs (Matsui and Corey, 2017). Recently, targeting miRNAs using chemical small molecules has become a promising strategy for disease treatment (Fan et al., 2019). We previously reported that Rb2 can bind to miR-216a specifically (Chen et al., 2021), and in this further study, we showed that Rb2 decreased the expression of endogenous miR-216a in macrophages and exerted the inhibitory role in M1 macrophages polarization and lipid deposition by targeting miR-216a.

Rb2 is one of the most highly abundant components in ginseng and has been reported to possess various bioactivities including anti-inflammatory, anti-oxidative, and anti-tumor effects (Fan et al., 2020). There are some reports that Rb2 may regulate cell death. Kim et al. (2019) found that Rb2 possesses neuroprotective properties that suppress the glutamate-mediated oxidative stress and neuronal cell death in HT22 hippocampal mouse neuron cells. Gao et al. (2015) reported that Rb2 has anti-apoptotic properties in dexamethasone-treated murine bone marrow–derived mesenchymal stem cells by the modulation of GPR120 expression. On the other hand, Rb2 has been found to have a marked cytotoxic effect and anti-proliferative activity against the human colon cancer cell line in a dose- and time-dependent manner (He et al., 2012). As for the atherosclerosis study, Rb2 ameliorates the atherosclerotic responses such as LPS-induced inflammation, apoptosis, and endoplasmic reticulum stress in endothelial cells and THP-1 cells via the AMPK-mediated pathway (Sun et al., 2020). In this study, we did not observe a significant influence on cell death of THP-1 macrophage cells induced by PMA. One possible explanation is that Rb2 may exert the differential effects on cell death due to various cell types and stimulations.

There are several reports involving microRNA-216a in the regulation of apoptosis and necrosis but with different effects under the condition of different diseases. For example, miR-216a overexpression can promote breast cancer cell apoptosis by targeting protein kinase C alpha (PKCα) (Cui et al., 2019) and increase cell apoptosis in response to gemcitabine in the pancreatic cancer cells (Zhang et al., 2017). By contrast, miR-216a inhibited neuronal apoptosis in a cellular Parkinson’s disease model (Yang et al., 2020), and alleviated LPS-induced apoptosis of the human lung carcinoma cell line A549 via regulating NF-κB signaling (Kong et al., 2020). Our previous studies showed that miR-216a can promote M1 macrophage polarization, lipid accumulation, and senescence through the Smad3/IκBα signaling pathway (Yang et al., 2019). Here, we revealed that miR-216a can inhibit macrophages apoptosis, and the underlying mechanisms need further investigation.

Cells in the atherosclerotic lesions can die in several ways, and the inflammatory response to each form of cell death is highly variable. In contrast to homeostatic apoptosis, highly pro-inflammatory necrotic types of cell death, such as necroptosis and pyroptosis tend to trigger an inflammatory response. Inhibition of the potential triggers of necrosis or of key proteins in the necrotic pathway can ameliorate the severity of atherosclerosis disease (Martinet et al., 2011). Pyroptosis and necroptosis lead to disruption of the plasma membrane and release of the cellular contents and various DAMPs. Atherosclerotic plaques are populated mostly by macrophages, which equipped with a set of pattern recognition receptors, including Toll-like and scavenger receptors, readily respond to DAMPs (Frank and Vince, 2019). Several studies have shown that inflammation caused by Toll-like receptor activation by endogenous ligands participates in the development of atherosclerosis (Serbulea et al., 2018; Zhou et al., 2020).

In this context, foam cells obtained by long-term culture in the presence of ox-LDLs may preferentially undergo pyroptosis combined with other types of necrotic death. The recent study pointed out that necrotic cell death pathways may serve as potential markers for atherosclerosis disease severity (Karunakaran et al., 2016). These phenomena have not been previously observed in traditional cellular models using one dose of ox-LDLs and subsequent culture for 3 days. Thus, it is tempting to speculate that in long-term cultures (and probably also in foam macrophages in advanced atherosclerotic plaques), switching between cell death pathways is an important effector mechanism of miR-216a.

Macrophages display phenotypic heterogeneity and plasticity in atherosclerotic plaques, mainly including two kinds of subsets: M1 proinflammatory subtype and M2 anti-inflammatory subtype. M1 macrophages are mostly abundant in unstable plaques and promote atherosclerosis progression by secreting pro-inflammatory cytokines MCP-1 and TNFα (Chinetti-Gbaguidi et al., 2015). In the present study, we found that Rb2 attenuated atherosclerosis plaque progression and instability induced by miR-216a by inhibiting M1 pro-inflammatory polarization and lipid accumulation in the mice model. In *in vitro* experiments, we found that Rb2 suppressed the TNFα and MCP-1 mRNA expression by neutralizing miR-216a in THP-1–derived macrophages. Moreover, Rb2 was found to restore the protein expression of Smad3 and the IκBα pathway, and decreased telomerase activity by counteracting miR-216a.

THP-1 is an immortalized monocyte-like cell line with homogeneous genetic/epigenetic backgrounds and purity of the macrophage population. As opposed to the primary monocytes or macrophages, THP-1 cells differentially express cell surface receptors (Bosshart and Heinzelmann, 2016). Also, differentiation of THP-1 cells induced with various stimuli (e.g., PMA) is not completely comparable to the primary monocyte-derived macrophages (Shiratori et al., 2017; Tedesco et al., 2018). Therefore, THP-1 may not be an adequate model to study plaque macrophages or monocyte-derived macrophages and foam cells, and this is the limitation of our study. Given that human primary monocytes or macrophages are thought to be an adequate model to study atherosclerotic plaque, our study further confirmed the effect of Rb2 on lipid accumulation in the human PBMC-derived macrophages and foam cells, which showed similar results as in the THP-1 model.

In summary, our study provided new evidence that Rb2 inhibited M1 polarization, lipid accumulation, and atherosclerosis plaque progression by counteracting miR-216a through the Smad3/IκBα pathway. The findings indicated that Rb2 might be a potential therapeutic molecule for atherosclerosis progression by targeting miR-216a. Given that chronic (long-term) accumulation of foam cells in the intima takes place in the constant presence of proatherogenic ox-LDLs, the role of Rb2 in atherosclerosis progression needs further investigation.

## Data Availability

The original contributions presented in the study are included in the article/Supplementary Material, further inquiries can be directed to the corresponding author.
