# Supplementary material for: Ginsenoside Rb2 Alleviated Atherosclerosis by Inhibiting M1 Macrophages Polarization Induced by MicroRNA-216a
Source: Front Pharmacol. 2022 Jan 3;12:764130. doi: 10.3389/fphar.2021.764130 (PMC8762269; doi:10.3389/fphar.2021.764130)
Supplement: Supplementary file 1 [file DataSheet1.docx]

SUPPLEMENTARY MATERIAL

**Title:** Ginsenoside Rb2 alleviated atherosclerosis progression by inhibiting M1 macrophages polarization induced by microRNA-216a

**1 SUPPLEMENTARY METHODS**

**1.1 The Immunohistochemical Analyses**

The aortas of ApoE^-/-^ mice were collected to analyze the status of atherosclerosis plaque. Immunohistochemical staining was used to assess intraplaque macrophages and matrix metalloproteinase 9 (MMP-9) expression. Frozen sections were prepared and incubated with the primary antibodies for overnight at 4°C. The following primary antibodies (catalogue number and dilutions) were used: rabbit mAb CD68 (ab125212, 1:100, Abcam, USA), rat mAb CD16+32 (ab25235, 1:100, Abcam, USA) and rabbit mAb matrix MMP-9 (ab38898, 1:200, Abcam, USA). Then, the secondary antibodies goat polyclonal anti-rabbit IgG (PV-6001) and goat anti-rat IgG (PV-9004, ZSGO-BIO, Beijing, China) were respectively incubated 40 min at room temperature. The sections were visualized using DAB kit (ZSGO-BIO, Beijing, China). The sections were scanned by Pannororamic SCAN (3DHISTECH, Budapest, Hungary) and analyzed by Image-pro Plus 6.0 software.

**1.2 Dual Luciferase Reporter Assay**

Dual-luciferase reporter assay was used to assess the effects of Rb2 on the expression of Smad3, the direct target of miR-216a. PMIR-REPORT-Smad3-3’UTR luciferase plasmid was constructed according to our previous study (Yang et al., 2018). The plasmid containing wild type or mutant Smad3 3’UTR at the dose of 100 ng were co-transfected into human embryonic kidney cell HEK293T cells (China Infrastructure of Cell Line Resources, Beijing, China) with 50 nM microRNA-216a (miR-216a) mimics or negative control for 8 h. Then the cells were incubated with 10 μM ginsenoside Rb2 (Rb2) for 48 h. The luciferase activity was detected by the Dual-Luciferase Reporter Assay System (Promega, Madison, WI, USA) using an Infinite M200 Pro Multimode Microplate Reader (Tecan, Zurich, Switzerland).

**1.3 Telomerase Enzyme Activity Measurement**

The telomerase repeat amplification protocol (TRAP) assay was performed to assess the telomerase activity during macrophages differentiation and polarization (Hou et al., 2001; Yang et al., 2019). A total of 1×10^5^ cells were suspended in 50 μL CHAPS buffer (Roche, Mannheim, Germany) and lysed for 30 min on ice, and then centrifuged for 30 min at 16000 rpm at 4°C. The supernatant was used as template for RT-qPCR. The reaction was performed in 20 μL solution, including 0.5 U Hotstar DNA polymerase (Takara, Dalian, China), 200 nM TS primers, 100 nM Cxa primers, 0.4×SYBR-Green, 10×buffer and dNTP on the ABI 7500 System (Applied Biosystems, Massachusetts, USA).

The primers were as the following: TS primer 5’ AATCCGTCGAGAACAGTT 3’, Cxa primer 5’ GTGTAACCCTAACCCTAA CCC 3’. The telomerase activity was calculated by relative quantification (2^-ΔΔCt^) method.

**1.4 MicroRNA Expression Analysis**

Total RNA of macrophages was extracted by TRIzol reagent (Invitrogen, Carlsbad, CA, USA), and cDNA was reverse transcribed with All-in-one^TM^ miRNA First-Strand cDNA System kit (GeneCopoeia, Rockville,MD, USA). Next, the expression of miR-216a were assessed by All-in-one^TM^ miRNA qPCR Detection kit (GeneCopoeia, Rockville,MD, USA) on the ABI 7500 System. The expression of U6 small nuclear RNA was used as internal reference and fold changes were calculated by 2^-ΔΔCt^. The primers were as the following: miR-216a-5p primers 5’ TCTCAGCTGGCAACTGTGAAA 3’, U6 primer 5’ GGTCGGGCAGGAAAGAGGGC 3’.

**1.5 mRNA Expression Analysis**

Total RNA of macrophages was reverse transcribed using PrimeScript Reverse Transcriptase assay (Takara, Dalian, China). The mRNA expression was assessed by RT-qPCR using SYBR Green qPCR mix (YEASEN, Shanghai, China) in the ABI 7500 System. The expression of glyceraldehyde-3-phosphate dehydrogenase (GAPDH) was used as internal reference and fold changes were calculated by relative quantification (2^-ΔΔCt^). The gene primers are listed in Supplementary Table 1.

**1.6 Western Blot Analysis**

The western blot assay was performed to explore the effects of Rb2 on protein expression of Smad3 and nuclear factor kappa B inhibitor alpha (IκBα). Macrophage protein extracts were isolated with RIPA lysis buffer (Beyotime Biotechnology, Shanghai, China) containing protease inhibitor (Roche, Mannheim, Germany). Equal amount of samples were separated by 10% SDS-PAGE gels, then transferred to nitrocellulose membranes (Millipore, MA, USA). After blocking with 10% skimmed milk for 2 h, the membranes were incubated with primary antibodies overnight at 4°C.

The following Primary antibodies (catalogue number and dilutions) were Rabbit mAb Smad3 (12747, 1:1000) (Cell Signaling Technology, Danvers, MA, USA) and mouse mAb IκBα (9242, 1:1000) (Cell Signaling Technology, Danvers, MA, USA). Mouse mAb GAPDH (TA-08, 1:2000, ZSGB-BIO, Beijing, China) was applied as an internal reference. Then, the secondary antibodies anti-rabbit (7074, 1:5000, Cell Signaling Technology, Danvers, MA, USA) and anti-mouse (ZB-2305, 1:5000, ZSGB-BIO, Beijing, China) antibodies were respectively incubated 1.5 h at room temperature. Bands were visualized with FluorChem R, M and E Systems (ProteinSimple, CA, USA) and quantified with AlphaView Software.

**1.7 Lipid Uptake Abilities**

The effect of Rb2 on lipid uptake ability of M1 macrophages were assessed by the Oil red O staining. Briefly, after miR-216a mimics transfection and Rb2 treatment, macrophages were incubated with 50 μg/mL oxidized low-density lipoprotein (ox-LDL) (Yiyuan Biotech, Guangzhou, China) for 12 h at 37°C to transformed foam cells. After washing three times with Phosphate Buffered Saline (PBS), cells were stained with 0.3% Oil red O for 3 min and counterstained with hematoxylin (Leagene, Beijing, China) for 10 s at room temperature. The lipid accumulation status of cells was taken by light microscopic examination (Leica DM6000B). The ratio of lipid deposit was calculated as the percentage of stained to total cell area.

**1.8 Cholesterol Efflux Assay**

The Cholesterol efflux assay was performed as described previously (Traves et al., 2007). The macrophages were incubated with 5 μg/mL [^3^H]-cholesterol (Invitrogen, Carlsbad, CA, USA) and 50 μg/mL acetylated LDL (Yiyuan Biotech, Guangzhou, China) for 12 h, and then cultured in RPMI Medium Modified containing 0.2% fatty acid-free bovine serum albumin (Sigma-Aldrich, Saint Louis, MO, USA) for 4 h. After that, the cell supernatant was collected, and the cells were lysed by 0.1% Triton. Subsequently, the fluorescence intensity of supernatant and cell lysate were detected on Infinite M200 Pro Multimode Microplate Reader at excitation 469 nm and emission 537 nm. The cholesterol efflux rate was calculated as a percentage of fluorescence intensity in the supernatant, compared with total [^3^H]-cholesterol in the supernatant and cell lysate.

**1.9 Flow Cytometry**

The effect of Rb2 on miR-216a-mediated macrophages polarization was assessed by flow cytometry. After miR-216a mimics transfection and Rb2 treatment, the macrophages were digested by trypsin and collected by centrifuging for 5 min at 1000 rpm at 4°C. Then cells were resuspended by 300 µL 1×binding buffer with a density of 2-5×10^5^ /mL. Cells were stained with 5 µL fluorochrome-tagged monoclonal antibodies (Biolegend, San Diego, CA, USA) against CD86 (Allophycocyanin, APC) to typify the M1 phenotype, and against CD206 (P-phycoerythrin, PE) to characterize the M2 phenotype for 30 min. Accuri C6 Flow Cytometer (BD Biosciences, San Jose, CA, USA) was used to analyze the macrophages polarization.

The cell apoptosis or death were determined by flow cytometry with Annexin V/PI detection kit (BD Biosciences, New Jersey, USA) according to the manufacturer’s protocol. After trypsin digestion, foam cells were collected by centrifuging for 5 min at 1000 rpm at 4°C and resuspended by 300 µL binding buffer. A total of 5 µL fluorescein isothiocyanate (FITC) Annexin V was added into cell suspension, and this mixture was incubated for 15 min at room temperature. Subsequently, cell suspension was added 5 µL Propidium Iodide (PI) and incubated for another 5 min. The apoptosis status was detected by Accuri C6 Flow Cytometer.

**1.10 Senescence-Associated β-Galactosidase Staining**

The senescent status of macrophage was assessed by in situ staining for senescence-associated β-galactosidase (SA-β-gal) with staining kit (Beyotime Biotechnology, Shanghai, China) according to the manufacturer’s protocol. In brief, cells cultured in 12-well plates were fixed in 4% paraformaldehyde solution at room temperature for 15 min. Then the cells were incubated with fresh β-galactosidase staining solution (pH 6.0) for 16 h in a 37°C incubator without CO_2_. Photos of cells senescent staining were recorded by microscope and more than five different microscopic fields were acquired per sample. The percentages of SA-β-gal-positive cells were calculated by counting 5 fields.

# 2 SUPPLEMENTARY TABLES AND FIGURES

The supplemental materials included 2 Tables and 3 Figures.

**Supplementary Table 1**. The primers for quantitative RT-qPCR.

**Supplementary Table 2.** Lipid profiles of the ApoE^-/-^ atherosclerotic mice.

**Supplementary Figure 1.** The cell viability of THP-1 cells during PMA treatment and transfection.

**Supplementary Figure 2.** The effect of Rb2 on THP-1-derived foam cell death.

**Supplementary Figure 3.** The effect of Rb2 on THP-1 derived macrophages apoptosis and necrosis.

**Supplementary Table 1**. **The primers for quantitative RT-qPCR.**

| **Species** | **Gene** |  | **Sequence (5’ −> 3’)** |
| --- | --- | --- | --- |
| Mice | *ABCG1* | forward | GCTACATCATGCAGGACGA |
|  |  | reverse | AGGGCTGTCAGGATCTCTTT |
|  | *ABCA1* | forward | GCTGTGGTGACATGGCTTGTT |
|  |  | reverse | GGTAGCTCAGGCGTACAGAGA |
|  | *GAPDH* | forward | TCTCCTGCGACTTCAACA |
|  |  | reverse | TGTAGCCGTATTCATTGTCA |
| Human | *TERT* | forward | CTGCGTTTGGTGGATGATTTCT |
|  |  | reverse | GCCTCGTCTTCTACAGGGAAGTT |
|  | *TNFα* | forward | CCGAGTGACAAGCCTGTA |
|  |  | reverse | GGACCTGGGAGTAGATGAG |
|  | *MCP1* | forward | CAAACTGAAGCTCGCACTCTCGCC |
|  |  | reverse | ATTCTTGGGTTGTGGAGTGAGTGTTCA |
|  | *CD36* | forward | TGATGAACAGCAGCAACA |
|  |  | reverse | CACAGCCAGATTGAGAACT |
|  | *SR-A1* | forward  reverse | GATGCTCGCTCAATGACA  GGTGGTGTTCTTCCTCATT |
|  | *ABCG1* | forward  reverse | CGGCTTCCTCTTCTTCTC  CCAGTAGTTCAGGTGTTCC |
|  | *p21*  *p16* | forward  reverse  forward  reverse | GAGCAGGCTGAAGGGTCCCCAGGT  GCTTCCTGTGGGCGGATTAGGGCT  GCCCAACGCACCGAATAGTTACG  CACCACCAGCGTGTCCAGGAA |
| Human | *GAPDH* | forward  reverse | GAAGGTGAAGGTCGGAGTCA  GGAAGATGGTGATGGGATTTC |

Abbreviations: *ABCA1*, ATP binding cassette subfamily A member 1; *ABCG1*, ATP binding cassette subfamily G member 1; *GAPDH*, glyceraldehyde-3-phosphate dehydrogenase; *TERT*, telomerase reverse transcriptase; *TNFα*, tumor necrosis factor; *MCP1*, C-C motif chemokine ligand 2; *CD36*, CD36 molecule; *SR-A1*, macrophage scavenger receptor 1.

**Supplementary Table 2. Lipid profiles of the ApoE^-/-^ atherosclerotic mice.**

|  | **Ad-NC**  **(n=6)** | **Ad-miR-216a**  **(n=5)** | **Ad-NC+Rb2**  **(n=7)** | **Ad-miR-216a+Rb2**  **(n=5)** | ***P**** | ***P* #** |
| --- | --- | --- | --- | --- | --- | --- |
| Triglycerides, mmol/L | 1.3±0.2 | 1.7±0.2 | 1.3±0.2 | 1.5±0.2 | 0.130 | 0.486 |
| Total cholesterol, mmol/L | 25.7±1.9 | 31.2±2.7 | 29.2±2.6 | 28.6±3.5 | 0.171 | 0.530 |
| HDL cholesterol, mmol/L | 6.3±0.5 | 7.0±0.4 | 7.3±0.7 | 6.8±0.5 | 0.422 | 0.797 |
| LDL cholesterol, mmol/L | 19.4±1.5 | 24.2±2.4 | 22.0±1.9 | 21.8±3.0 | 0.139 | 0.479 |

Abbreviations: ApoE^-/-^ mice indicates apolipoprotein E-deficient mice; HDL, high-density lipoprotein; LDL, low-density lipoprotein.

**P*, Ad-miR-216a vs. Ad-NC; ^#^*P*, Ad-miR-216a+Rb2 vs. Ad-miR-216a.


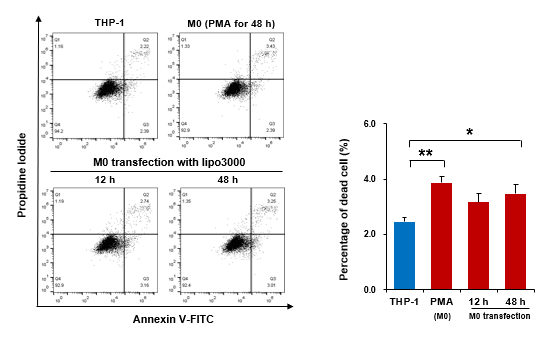


**Supplementary Figure 1. The cell viability of THP-1 cells during PMA treatment and transfection.**

Representative flow cytometry images and quantification of cell death in the THP-1 with PMA treatment and macrophages with transfection for 12 h and 48 h (n=3 for each group).

**P*<0.05, ***P*<0.01, compared to THP-1 group.

**
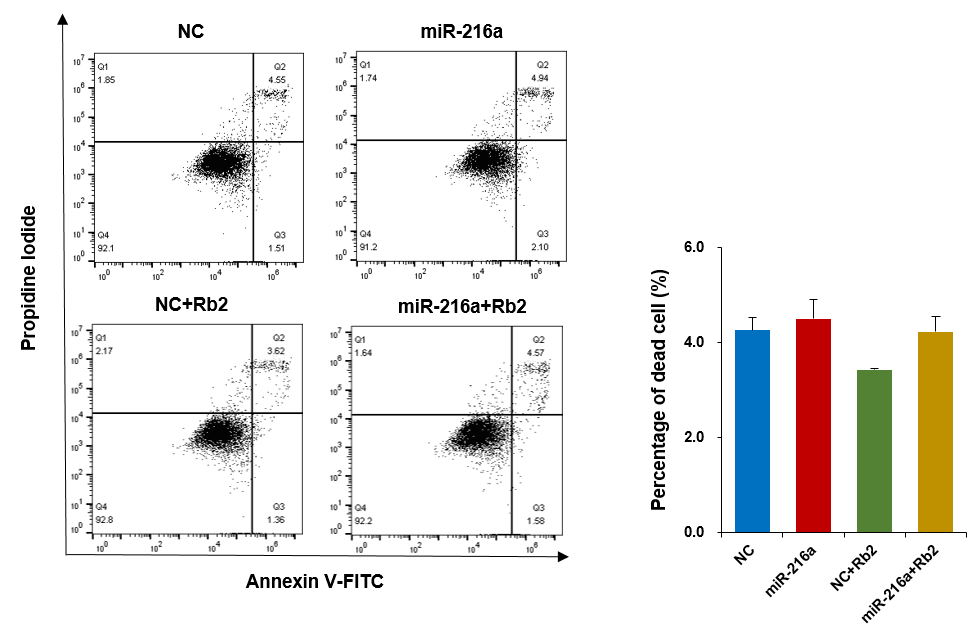
**

**Supplementary Figure 2. The effect of Rb2 on THP-1-derived foam cell death.**

Representative flow cytometry images and quantification of cell death with or without Rb2 treatment in the THP-1 derived foam cells transfected with miR-216a mimics (n=3 for each group).


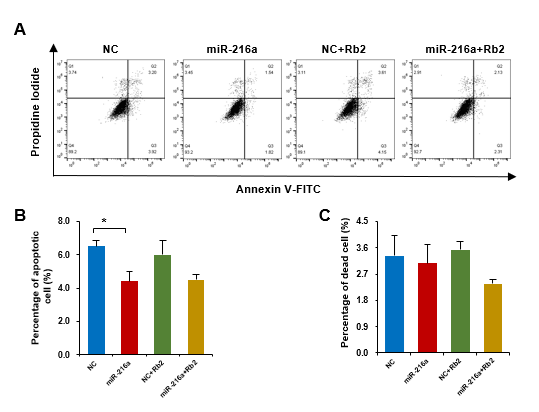


**Supplementary Figure 3. The effect of Rb2 on THP-1 derived macrophages apoptosis and necrosis.**

1. Representative flow cytometry images of cell apoptosis and death with or without Rb2 treatment in macrophages transfected with miR-216a mimics (n=3 for each group).
2. Quantification of the percentage of apoptotic cell with or without Rb2 treatment in macrophages transfected with miR-216a mimics (n=3 for each group).
3. Quantification of the percentage of dead cell with or without Rb2 treatment in macrophages transfected with miR-216a mimics (n=3 for each group).

**P*<0.05, compared to NC group.

# 3 Reference

Hou, M., Xu, D., Bjorkholm, M., and Gruber, A. (2001). Real-time quantitative telomeric repeat amplification protocol assay for the detection of telomerase activity. *Clin. Chem.* 47, 519-24.

Traves, P. G., Hortelano, S., Zeini, M., Chao, T. H., Lam, T., Neuteboom, S. T., et al. (2007). Selective activation of liver X receptors by acanthoic acid-related diterpenes. *Mol. Pharmacol.* 71, 1545-53. doi: 10.1124/mol.106.031906.

Yang, S., Li, J., Chen, Y., Zhang, S., Feng, C., Hou, Z., et al. (2019). MicroRNA-216a promotes M1 macrophages polarization and atherosclerosis progression by activating telomerase via the Smad3/NF-kappaB pathway. *Biochim. Biophys. Acta Mol. Basis. Dis.* 1865, 1772-1781. doi: 10.1016/j.bbadis.2018.06.016.

Yang, S., Mi, X., Chen, Y., Feng, C., Hou, Z., Hui, R., et al. (2018). MicroRNA-216a induces endothelial senescence and inflammation via Smad3/IkappaBalpha pathway. *J. Cell Mol. Med.* 22, 2739-2749. doi: 10.1111/jcmm.13567.
